# Integer Charge Transfer Model–PTCDA on MgO(001)/Ag(001) Probing the Transition from Single to Double Integer Charge Transfer

**DOI:** 10.1021/acs.jpcc.4c08104

**Published:** 2025-01-08

**Authors:** Philipp Hurdax, Michael Hollerer, Christian S. Kern, Peter Puschnig, Martin Sterrer, Michael G. Ramsey

**Affiliations:** Institute of Physics, NAWI Graz, University of Graz, Universitätsplatz 5, 8010 Graz, Austria

## Abstract

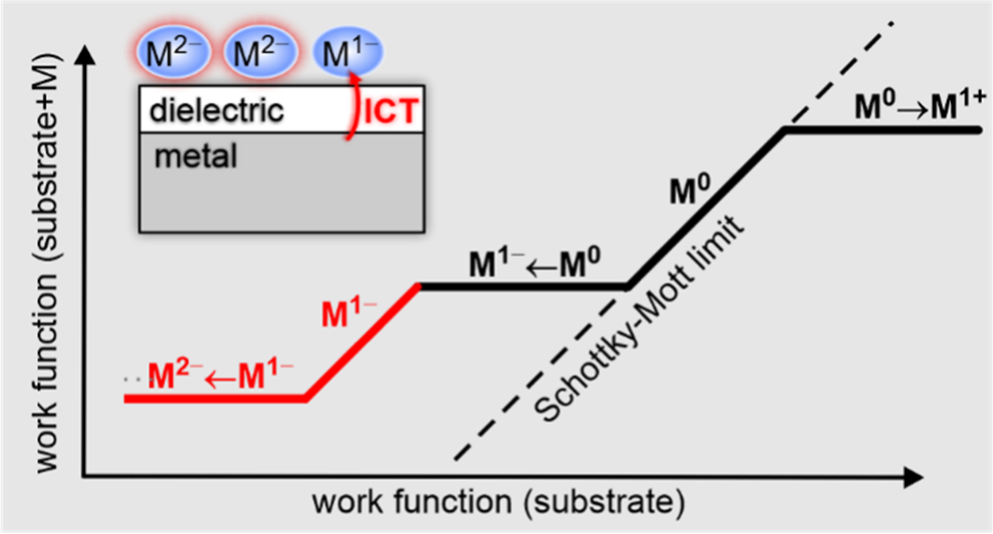

For weakly interacting adsorbate/substrate systems, the integer charge transfer (ICT) model describes how charge transfer across interfaces depends on the substrate work function. In particular, work function regimes where no charge transfer occurs (vacuum level alignment) can be distinguished from regions where integer charge transfer by electron tunneling from substrate to adsorbate or vice versa takes place (Fermi level pinning). While the formation of singly integer charged molecular anions and cations of organic semiconductors on various substrates has been well described by this model, the double integer charging regime has so far remained unexplored and experimentally elusive. Here, we extend the integer charge transfer model to the transition from single to double integer charging. This was made possible by combining a molecular adsorbate with high electron affinity (Perylenetetracarboxylic-dianhydride (PTCDA)) with a substrate with tunable work function (ultrathin MgO(001) films on Ag(001)). Our results, obtained with scanning tunneling microscopy (STM), photoemission spectroscopy (PES), work function measurements and density function theory (DFT) calculations, show that after completing the single negative charging of all molecules in a PTCDA monolayer in the first Fermi level pinning regime, the system transitions to a vacuum level alignment regime for singly charged molecules when the substrate work function is reduced, and finally enters the second Fermi level pinning regime at very low substrate work function, in which the molecules become doubly negatively charged.

## 1 Introduction

The type of interaction and the alignment of the energy levels at the interfaces between solid substrates and various adsorbates such as metal atoms and particles, molecules and molecular monolayers, or thin polymer films, controls the electronic and chemical properties of materials. Their detailed knowledge is required for understanding processes in catalysis, electronics or sensor technology. Charge transfer across interfaces often occurs in the case of strong chemical interaction, but also for weakly interacting systems bonded by van der Waals forces, charge transfer can take place if the energetic position of the electron accepting or donating levels in the adsorbate relative to the Fermi energy of the substrate allows this. Especially for weakly interacting interfaces between passivated metal substrates and polymer films, which occur in organic electronic devices, the integer charge transfer (ICT) model was introduced.^1^ It describes the transition between regimes where no charge transfer (vacuum level alignment) and charge transfer (Fermi level pinning) take place and is based on the relative energy level alignment.^2−4^ While the transferred charge is considered to lead to the formation of polarons in polymers,^5^ it results in the creation of anions or cations in adsorbed single molecules or molecular monolayers. The ICT model is thus of relevance also for the fundamental understanding of charge transfer into or out of molecules adsorbed on metal–supported ultrathin dielectrics including well-ordered oxides, halogenides, chalcogenides, graphene, and other two-dimensional (2D) materials, which can be investigated on a molecular level using surface science methods. In this work, we use a model system consisting of Perylenetetracarboxylic-dianhydride (PTCDA) monolayers grown on ultrathin MgO(001) thin films on Ag(001) to extend the ICT model from the well-studied single integer charging regime to the so far unexplored double integer charging regime.

Traditionally, ultrathin dielectric layers on metals have been regarded as purely passive decoupling layers. For instance, this has allowed the imaging of frontier molecular orbitals with scanning tunneling microscopy (STM) by decoupling the molecular wave function from the metal substrate,^6^ to increase the magnetic remanence in single atom magnets,^7^ or to study the charge state lifetimes of single molecules.^8^ Ultrathin layers of large band gap oxides have also been successfully employed as substrates to create surface science models of heterogeneous catalysts.^9,10^ However, work in fields related to both catalysis and organic electronics has shown that the presence of ultrathin dielectrics can also actively promote charge transfer.^11,12^ When either the highest occupied or the lowest unoccupied adsorbate state (HOMO and LUMO) aligns with the Fermi level of the substrate, charge transfer takes place through quantum mechanical tunneling. This recognition traces back to the work of G. Pacchioni′s group, who predicted that some ultrathin oxide layers on metals favor charge transfer to adsorbed metal atoms by lowering the substrate work function.^11,13,14^ Early experimental studies have then inferred charge transfer on a local level from the appearance of individual atoms and clusters in STM images.^15,16^

**Figure 1:**
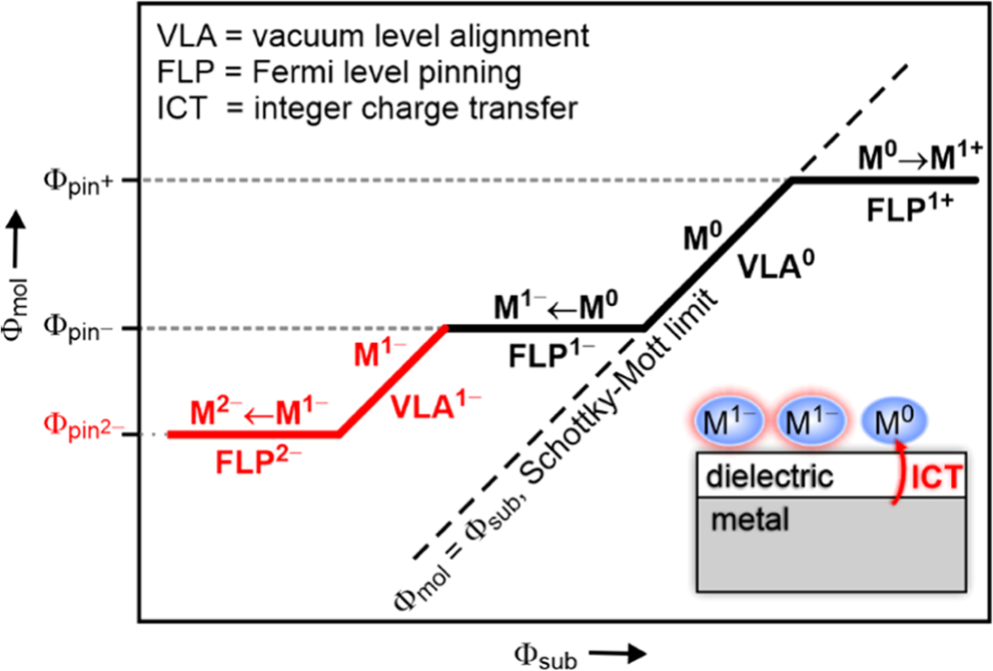
Integer charge transfer model. Schematic plot of the dependence of the work function (Φ_mol_) of the substrate after adsorption of a molecular adsorbate (M) on the variation of the work function of the clean substrate (Φ_sub_). Lines with a slope of 1 represent vacuum level alignment (VLA) regimes. Lines with a slope of 0 are the Fermi level pinning (FLP) regimes.

On a global level, for polymer and molecular films of interest to organic electronics, be it on metals or on dielectric thin films, charge transfer has been understood to be reflected in the distinct behavior of the work function after molecular film growth (Φ_mol_) as a function of the work function of the pristine substrate (Φ_sub_), sometimes called the Mark of Zorro curve.^1^ At the center of the curve, representing the region where no charge transfer occurs, the resulting work function (Φ_mol_) shifts with the work function of the substrate (Φ_sub_) at a slope of 1 (Φ_mol_ = Φ_sub_, Figure 1). On either side of this vacuum level alignment (VLA^0^) regime, a Fermi level pinning (FLP) regime is encountered, with Φ_mol_ assuming a constant value (pinning work function, Φ_pin_). This phenomenon has been understood to arise from charge transfer between the substrate and the molecular film in either direction, leading to the formation of molecular anions (FLP^1–^) or cations (FLP^1+^).

On metals, hybridization of molecular states with the substrate allows electronic equilibration to be achieved by fractional charge transfer affecting all molecules in the molecular monolayer equally.^17^ By contrast, on weakly interacting substrates such as dielectric thin films, hybridization between molecular and substrate states is strongly attenuated, and instead, integer charge transfer occurs through electron tunneling. Recently, the equilibration process of FLP on dielectric interlayers has been shown to result from a mix of singly charged and uncharged molecules in the first monolayer (ML).^18,19^ The number of charged molecules required to reach Φ_pin_ for a molecule with electron affinity EA is determined by Φ_sub_, which implies that at some value of Φ_sub_, all molecules will be singly integer charged.

Evidence for the ICT model has been obtained with a variety of different substrate/adsorbate combinations, clearly demonstrating the transition from VLA to FLP regimes for both, the formation of anions (FLP^1–^) and cations (FLP^1+^),^20^ and in some cases even covering the entire range from FLP^1–^ to FLP^1+^.^21^ This has been achieved through the use of different substrate materials that allow the required large range of Φ_sub_ to be covered. However, while there are examples where the affinity level (LUMO) of molecules adsorbed on metals is found significantly below the Fermi level,^22,23^ suggesting full (double) occupation of the previously empty LUMO, on dielectric substrates, where the ICT mechanism holds, the transition from single to double integer charging of molecular adsorbates has not been observed so far. As shown in Figure 1 for the case of anion formation, once all molecules are individually integer charged, a second VLA regime is expected (VLA^1–^), which then transitions to the FLP of dianions (FLP^2–^) (Figure 1, red part). The second VLA regime exists because once all molecules are singly integer charged, resulting in a split of the former LUMO into a singly occupied and singly unoccupied molecular orbital (SOMO and SUMO), an additional energetic contribution is required to overcome the Coulomb barrier for double occupation of the orbital. It is clear from Figure 1 that this regime can only be achieved by the combination of a substrate with a sufficiently low work function with a molecular adsorbate with a high EA.

Here we address the question if double integer charging of molecules on weakly interacting substrates is indeed possible and if the integer charge transfer model is extendable to the double charging regime. For this, we employ a substrate system consisting of ultrathin MgO(001) films grown on a Ag(001) substrate, whose work function has previously been shown to be tunable in a wide range (2.5–4.5 eV).^18^ As molecular adsorbate, we choose PTCDA due to its high electron affinity of ∼3.5 eV in the bulk phase.^24,25^ Our results, obtained through a combination of STM and angular resolved ultraviolet photoemission spectroscopy (ARUPS) experiments, and density functional theory (DFT) calculations, provide a clear picture of the charging regime beyond single integer charge transfer.

## 2 Methods

### 2.1 Experimental Details

The experiments were performed under ultrahigh vacuum (UHV) conditions in two separate setups, each equipped with metal and molecule evaporators for controlled deposition, a sputter gun for sample cleaning, and a low energy electron diffraction (LEED) apparatus for checking the surface crystallinity. The sample was heated by electron beam heating and the temperature was measured with a type-K thermocouple. The Ag(001) crystal was cleaned by cycles of Ar^+^ sputtering and annealing to 800 K. MgO(001) films were grown by Mg evaporation in an oxygen environment. Mg fluxes used were of the order of 1 Å/min as monitored by a quartz microbalance. The MgO deposition was done at a temperature of 540 K–600 K and at an O_2_ pressure of 1 × 10^–6^ mbar. MgO deposition was followed by slow cooling (roughly 2.5 K/min). The work function of the MgO(001)/Ag(001) samples could be reduced by annealing in UHV or further Mg exposure while annealing, and increased by O_2_ exposure (5 × 10^–7^ to −2 × 10^–4^ mbar) at moderate temperatures. Perylenetetracarboxylic dianhydride (PTCDA) was deposited at a rate of 1.5–3 Å/min at room temperature. As it was not possible to desorb PTCDA and reobtain clean MgO(001)/Ag(001), each data point in the work function diagrams represents a fresh MgO film preparation with the work function tuned to a different value. Angle-resolved UV photoemission spectroscopy (ARUPS) measurements were performed using a goniometer-mounted VG ADES 400 spectrometer equipped with a helium gas discharge lamp (Helium I, photon energy = 21.2 eV, angle of incidence = 60°). Work functions were extracted from the secondary electron cutoff in photoemission, which has been measured in a sample bias (−10 V) configuration. Binding energies (BE) are defined relative to the Fermi level (*E*_F_). UPS spectra shown were taken at room temperature in the experimental geometry that yielded the maximum photoemission intensity of the LUMO (polar angle of 50° along [110] with an angular acceptance of 1°). STM measurements were performed at 77 K with a Createc low-temperature STM attached to an ultrahigh-vacuum preparation chamber (base pressure 2 × 10^–10^ mbar), using electrochemically etched tungsten tips. The bias voltage (*V*_S_) was applied to the sample.

### 2.2 Computational Details

DFT calculations were performed with the Vienna ab initio simulation package (VASP).^26−29^ We modeled the interfaces in the repeated-slab approach with a minimum of 18 Å of vacuum between the slabs and a dipole correction to avoid spurious interaction between the slabs.^30^ The interfaces were represented by three layers of Ag, two layers of MgO and the molecular layer.

For interfaces with higher work function than the stoichiometric MgO layer, we placed 8, 12, and 16 interstitial oxygen atoms into the topmost Ag layer (32 atoms).^31^ All geometries were relaxed until the norm of all forces was below 0.005 eV/Å, where we used the PBE functional for exchange-correlation effects^32^ and the Tkatchenko-Scheffler method^33^ with iterative Hirshfeld partitioning^34^ and optimized van der Waals parameters^35^ in order to account for dispersive interactions. For the geometry relaxation, we used a 4 × 4 × 1 Γ-centered mesh to sample the Brillouin zone and a kinetic energy cutoff of 450 eV in conjunction with the projector-augmented wave method.^36^ For the density of states calculations, we used the same parameters as above, albeit a 8 × 8 × 3 *k*-mesh and 500 eV kinetic energy cutoff.

## 3 Results and Discussion

**Figure 2:**
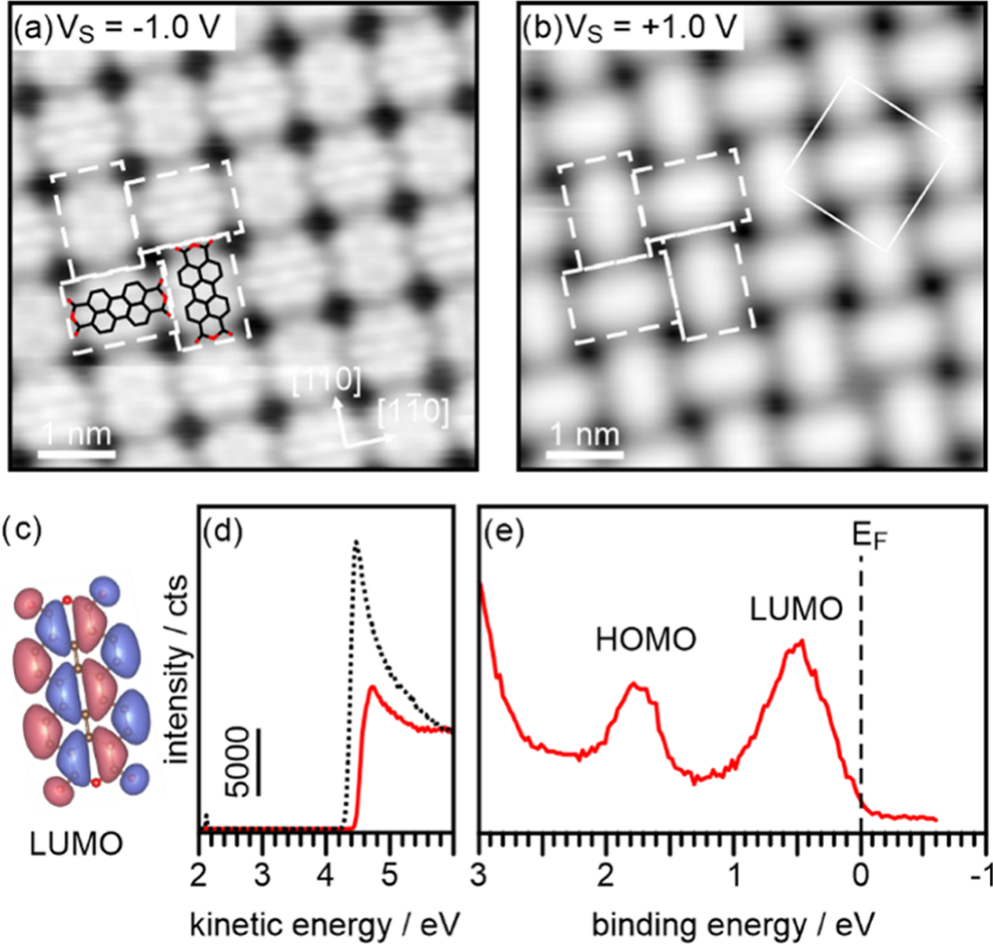
STM images of a PTCDA monolayer on Ag(001) taken at a bias voltage of (a) *V*_S_ = −1.0 V (filled states) and (b) *V*_S_ = +1.0 V (empty states). The T-type arrangement of the PTCDA molecules within the monolayer and the superstructure unit cell are indicated. The appearance of submolecular contrast in (a) exhibiting the general shape of the PTCDA LUMO shown in (c) suggests charge transfer into the PTCDA molecules on this surface. Work function measurements (cutoff spectra in (d)) reveal a small increase of the work function of clean Ag(001) (black dotted line) upon formation of the PTCDA monolayer (red line). In the valence region (shown in e) two molecular emissions are observed, which are identified as the occupied LUMO and the HOMO of PTCDA.

Before presenting the results for the charge transfer into PTCDA on ultrathin MgO(001)/Ag(001) films, we discuss its geometric and electronic structure upon adsorption on the clean Ag(001) substrate. Because of its high electron affinity, PTCDA is negatively charged on most of the commonly used metal single crystal surfaces, except Au.^23^ The STM images in Figure 2a,b reveal the T-type arrangement of the PTCDA molecules on Ag(001).^37^ Additionally, in the filled states image at a bias voltage of −1.0 V (Figure 1a), submolecular contrast appears, which can be attributed to the molecular LUMO by comparison with the nodal structure of the gas-phase LUMO presented in Figure 2c. The appearance of the LUMO in filled states images suggests charge transfer from the metal substrate into the molecule. This is confirmed by the ARUPS spectrum shown in Figure 2d, where the peaks at 0.5 eV binding energy (BE) and 1.75 eV BE result from the emission of the occupied LUMO and the HOMO, respectively.^22^ Since the LUMO peak is found to be located completely below the Fermi level, double occupation of the LUMO can be expected. However, this does not necessarily mean that the PTCDA molecules are doubly negatively charged. Backdonation of charge from deeper lying molecular states, which are hybridized with substrate Ag states, can in total yield a reduced and fractional charge transfer for this substrate/adsorbate system.

A strong driving force for charge transfer on metal surfaces is provided by the electron push-back upon adsorption of the molecules. This effect can reduce the work function of the metal substrate by more than 1 eV, thereby shifting the LUMO energy level of the adsorbed molecule below the Fermi level. On the other hand, if charge transfer into the PTCDA occurs, a dipole is created that counteracts the push-back induced work function reduction. It is thus not surprising, that the work function change upon adsorption of PTCDA on Ag(001) is small.^22^ As shown by the secondary electron cutoff spectra presented in Figure 2d, the work function increases from 4.3 eV for the clean Ag(001) surface to 4.45 eV for a full ML PTCDA.

The structure and charge state of PTCDA has previously been also investigated for adsorption on ultrathin decoupling layers on metals and on semiconductors,^38^ where the electron push-back effect is basically absent. While no charge transfer is observed if PTCDA adsorbs on a graphene layer on Pt(111)^39^ or Rh(110),^40^ singly negatively charged PTCDA molecules appear on a thin NaCl(100) layer on Cu(111),^41^ on a h-BN layer on Ni(111),^42^ and on ZnO.^43^ Note that this behavior correlates with the work functions of the clean substrates, which are 4.8 and 4.5 eV for graphene on Pt(111)^44^ and Rh(110),^45^ and 4.0, 3.6, and 3.5 eV for the latter three cases, respectively.^42,43,46^ This suggests that double charging of PTCDA requires adsorption on a substrate with a work function of less than 3.5 eV.

**Figure 3:**
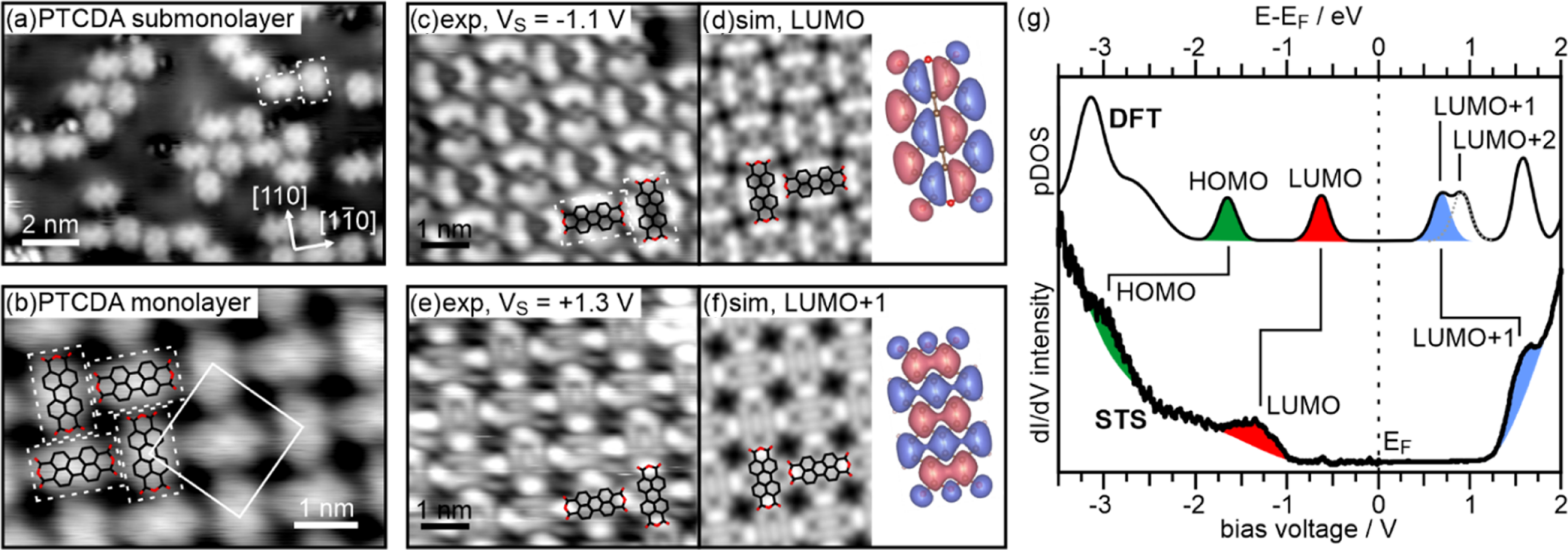
STM images of (a) submonolayer and (b) monolayer coverage PTCDA on MgO(001)/Ag(001). (a) 13 nm × 9 nm, *V*_S_ = −0.5 V; (b) 6 nm × 4 nm, *V*_S_ = +0.5 V. The T-type arrangement of the PTCDA molecules within the monolayer and the superstructure unit cell are indicated. (c) Filled-states (*V*_S_ = −1.1 V) STM image (6 nm × 5.4 nm) of a monolayer PTCDA on MgO(001)/Ag(001) and (d) corresponding simulated image for the energy range between 0 and −0.5 eV (*E* – *E*_F_) (see DOS in g) representing the STM contrast of the occupied LUMO of PTCDA. (e) Empty-states (*V*_S_ = +1.3 V) STM image of the same area as in (c), and (f) corresponding simulated image for the energy range between 0 and +0.75 eV (*E* – *E*_F_) (see DOS in (g)) representing the STM contrast of the unoccupied LUMO+1 of PTCDA. (g) (bottom) d*I*/d*V* spectrum taken above a PTCDA molecule within the monolayer and (top) calculated partial density of states (pDOS) of PTCDA on 2 ML MgO(001)/Ag(001) with an initial work function of Φ_sub_ = 2.96 eV.

Our own previous study of PTCDA on thin MgO(001) films on Ag(001) indicated the presence of charged molecules, with a significant bend of the molecular backbone.^47^ In the present study, we analyze the charge state of PTCDA on the MgO films in more detail by scanning tunneling microscopy and spectroscopy (STS), assisted by DFT calculations. The arrangement of PTCDA molecules in the submonolayer and monolayer regime on a 2–3 ML thin MgO(001) film is shown in the STM images in Figure 3a,b, respectively. We find the same T-type arrangement and an orientation of the molecules with their long molecular axis aligned along the high symmetry [110] directions as on Ag(001). The images in Figure 2a,b were obtained at bias voltages of +0.5 V (Figure 3a) and −0.5 V (Figure 3b), respectively, where the STM appearance resembles the geometric structure of the molecules. Imaging at both, more negative (Figure 3c), or higher positive (Figure 3e) bias completely changes the contrast, and a molecular orbital-like structure appears. An STS spectrum taken above a PTCDA molecule (Figure 3g, lower trace) reveals a large gap of 2.5 eV width around the Fermi level. In the filled states, two peaks appear at −1.3 and −2.8 V, while in the empty states a peak is observed at +1.5 V. To help identify the states from which these peaks originate, we present in Figure 3g also the calculated density of states (DOS) projected on the PTCDA molecule for PTCDA adsorbed onto a 2 ML thin MgO(001) film on Ag(001). Note that the work function of the MgO(001)/Ag(001) substrate in the calculation is 2.96 eV, which is similar to the expected work function of the stoichometric MgO(001)/Ag(001) film obtained in experiment employing the typical conditions for film preparation.

Similar to the STS spectrum, the calculated DOS has a gap around the Fermi energy (albeit with a smaller value due to the DFT self-energy error). The first states to appear below and above the Fermi level are the completely filled LUMO (below *E*_F_) and the empty LUMO+1 (above *E*_F_). Therefore, the calculations indicate double charge transfer into the molecule. With these results, the peaks observed in the STS spectrum at −2.8, −1.3, and +1.5 V can be assigned to the HOMO, LUMO, and LUMO+1 of PTCDA. For a direct comparison with the STM images in Figure 3c,e and further confirmation of this interpretation, we provide simulated STM images of the LUMO-derived state below the Fermi energy and the LUMO+1-derived state above the Fermi energy in Figure 3d,f, respectively. The remarkably good agreement between the experimental and simulated images additionally confirms the dianionic nature of the PTCDA molecules on the MgO(001) thin films in this experiment. Note that the different orbital appearances in the STM images below and above *E*_F_ in Figure 3c,e rule out the presence of singly negatively charged PTCDA molecules. In this case, the first empty state would also have LUMO character, since the singly occupied LUMO would then be split into a LUMO-derived SOMO and a LUMO-derived SUMO.^48^

**Figure 4:**
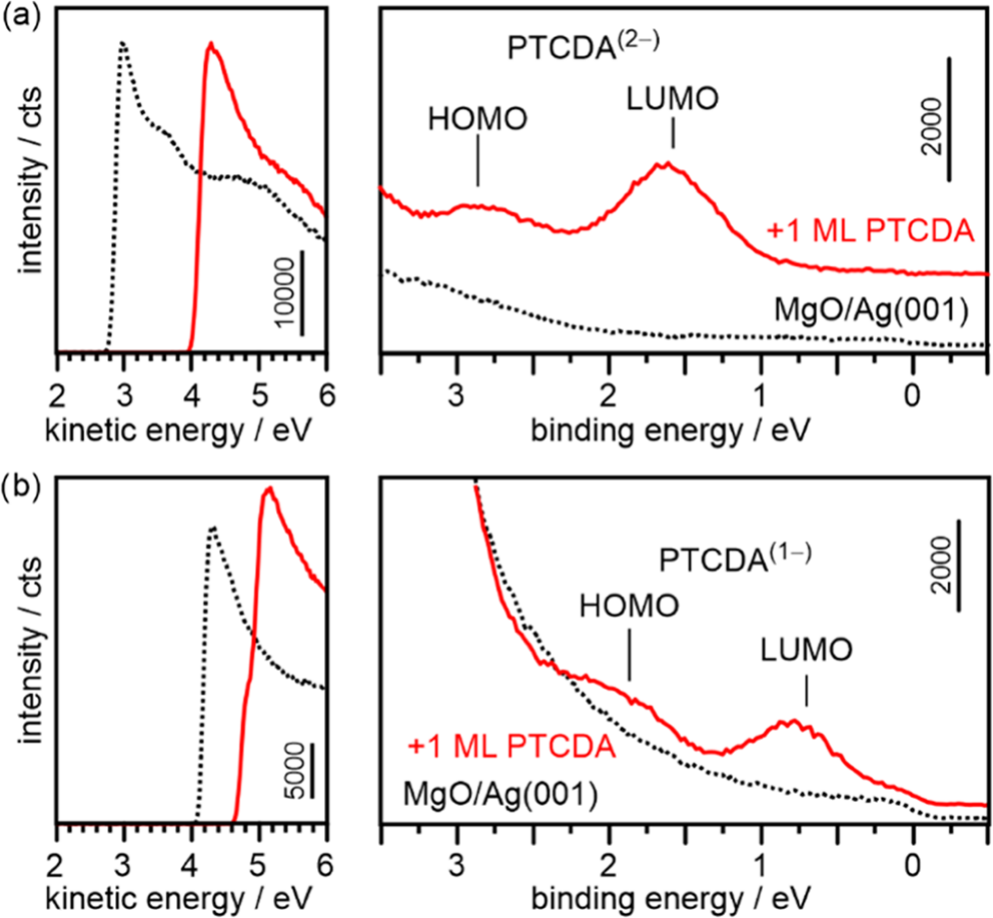
Secondary electron cutoff (left panels) and valence band region (right panels) of the pristine MgO(001)/Ag(001) thin film (black dotted lines) and of a monolayer of adsorbed PTCDA (red solid lines) for (a) low (Φ_sub_ = 2.82 eV) and (b) high (Φ_sub_ = 4.20 eV) initial substrate work function.

Both the experimental and computational results presented above show that PTCDA is doubly negatively charged if adsorbed on a MgO(001)/Ag(001) substrate with sufficiently low initial work function Φ_sub_. If the integer charge transfer model applies, the transition to a singly negatively charged PTCDA adsorbate is expected on increasing the work function. In this study, we have used the possibility to tune the work function of the MgO(001)/Ag(001) substrate by variation of the preparation parameters such as oxygen pressure and temperature.^18^ ARUPS has been employed to follow the evolution of the work function change and the electronic structure upon adsorption of a monolayer PTCDA. As examples, we present in Figure 4 results for low (Φ_sub_ = 2.82 eV) and high (Φ_sub_ = 4.20 eV) initial substrate work function, where the secondary electron cutoff region taken at normal electron emission angle is shown on the left, and the valence region taken in the geometry of the maximum emission from the PTCDA LUMO (at θ = 50° along [110], compare ref (47)) is shown on the right. Spectra of the clean substrate and of the PTCDA covered substrate are presented as black and red lines, respectively.

In the case of the sample with the low substrate work function of Φ_sub_ = 2.82 eV (Figure 4a), the adsorption of a ML PTCDA induces a large increase of the work function by ΔΦ = +1.22 eV (Φ_mol_ = 4.04 eV), consistent with charge transfer into PTCDA. Additionally, PTCDA-related emission features appear at binding energies (BE) with respect to the Fermi level of 2.8 and 1.6 eV, respectively. Through photoemission orbital tomography, these have been identified as emissions from the HOMO and LUMO of PTCDA, respectively.^47^ Their energies coincide with the STS peaks in Figure 3g and since the substrate work functions are similar in the two experiments, we assign them to the HOMO and fully occupied LUMO of the PTCDA dianions (PTCDA^(2−)^).

For the preparation with high substrate work function (Φ_sub_ = 4.20 eV, Figure 4b) the adsorption of a PTCDA ML results also in a work function increase, indicating charge transfer into PTCDA, however of smaller magnitude (ΔΦ = +0.62 eV, Φ_mol_ = 4.82 eV). Similarly to the low Φ_sub_ case, two peaks appear in the valence region, albeit at 1.80 and 0.75 eV BE. They can again be identified as emissions from the HOMO and the occupied LUMO of PTCDA, respectively. We tentatively assign these emissions to arise from singly negatively charged PTCDA such that the occupied LUMO is indeed the SOMO of the PTCDA^(1−)^ anions.

**Figure 5:**
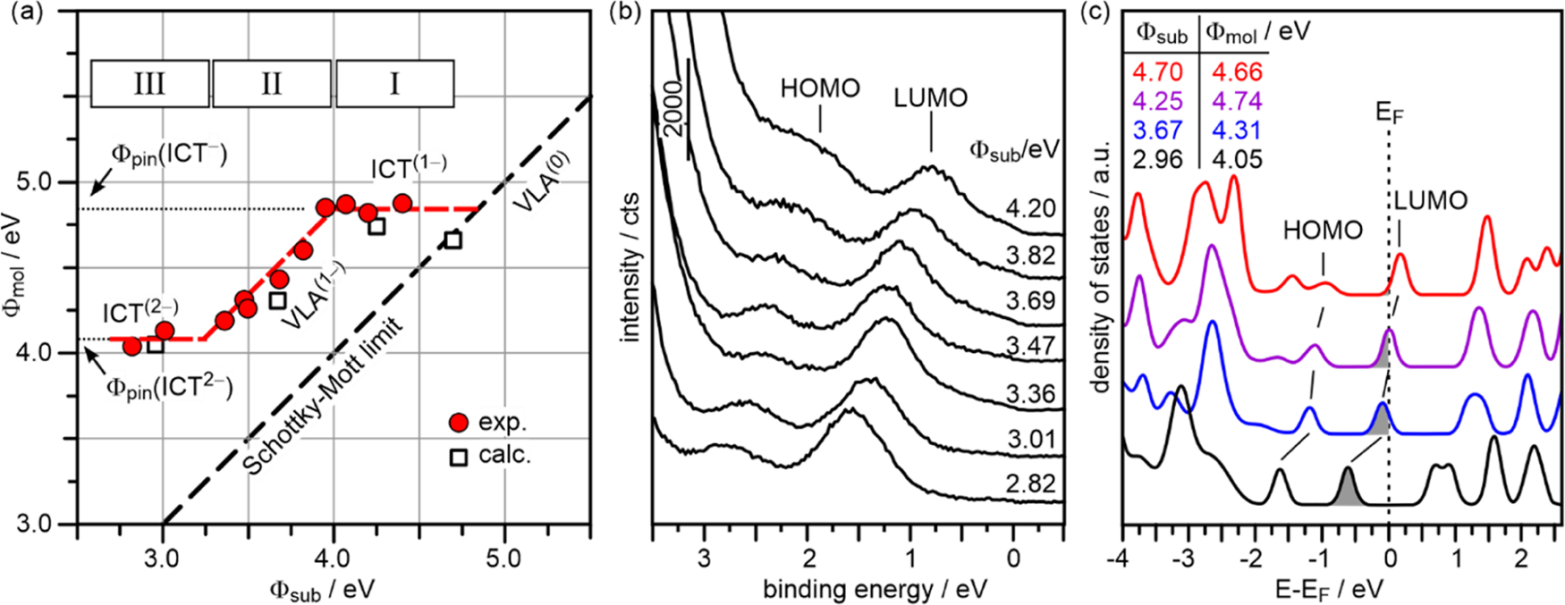
(a) Work function plot showing the dependence of the final work function (Φ_mol_) measured for the PTCDA/MgO(001)/Ag(001) layers on the initial substrate work function (Φ_sub_). The dashed black line with a slope of 1 represents the Schottky-Mott limit, which represents the vacuum level alignment of uncharged molecules (VLA^(0)^). Red circles are the experimental data points and white squares are results of calculations for different initial substrate work functions. The dashed red line is a guide to the eye. The experimental data points fall into three different regions: (I) Fermi level pinning of singly charged PTCDA (ICT^(−)^); (II) vacuum level alignment of singly charged PTCDA (VLA^(−)^); (III) Fermi level pinning of doubly charged PTCDA (ICT^(2−)^). (b) Valence-band UPS spectra of 1 ML PTCDA on MgO(001)/Ag(001) with different Φ_sub_. (c) Calculated molecule-projected density of states of PTCDA on 2 ML MgO(001)/Ag(001). The different colors represent different Φ_sub_. The Φ_sub_ values as well as the corresponding Φ_mol_ values are provided in the Figure.

While this interpretation is not immediately apparent from the results in Figure 4, the following analysis of the work function change induced by adsorption of PTCDA on MgO(001)/Ag(001) substrates covering a wide range of Φ_sub_ will confirm this assignment and reveal a conclusive picture of Φ_sub_ dependent charge transfer. As usual in ICT studies, in Figure 5a we plot the final work function after deposition of the molecular monolayer, Φ_mol_, against the initial substrate work function, Φ_sub_.

Over the entire accessible Φ_sub_ range, the ML of PTCDA causes an increase of Φ. All data points lie above the Schottky-Mott limit, indicating electron transfer to the molecular monolayers in all cases. The behavior of the work function of Figure 5a can be seen to consist of three regimes: For Φ_σub_ > 4.0 eV (I) and Φ_sub_ < 3.3 eV (III), we observe constant final work functions. These can thus be identified as Fermi level pinning regimes with, however, different pinning work functions of 4.8 and 4.1 eV, respectively. Between those regimes, for 3.3 eV < Φ_sub_ < 4.0 eV (II), the final work function shifts with the substrate work function, i.e., parallel to the Schottky-Mott limit, but 0.8 eV above it. Thus, region II can be considered a vacuum level alignment-like regime, albeit with singly charged molecules.

This behavior can be understood by considering the interplay of electrostatics and electron level alignment, if the possibility of dianion formation is considered. For very high work functions (Φ_sub_ > 4.8 eV), all molecules would remain neutral and follow the Schottky-Mott limit of vacuum level alignment, if there are no large contributions to the work function of the neutral PTCDA molecules, such as pushback or molecular dipole moments. On lowering Φ_sub_, which shifts the empty LUMO of the neutral PTCDA molecules toward and across the Fermi level, the first singly charged molecules would appear when Φ_sub_ ≤ 4.8 eV. Since integer charge transfer occurs, the occupied LUMO is split into a SOMO and a SUMO. This is the ICT^(−)^ region with the corresponding pinning work function for PTCDA anion formation of Φ_pin_(ICT^(−)^) = 4.8 eV. At Φ_sub_ = 4.0 eV, the entire ML consists of singly charged anions. On crossing region II, which is the vacuum level alignment regime for singly charged PTCDA anions, the energy levels of the anion are shifted with Φ_sub_. This is nicely reflected in the ARUPS spectra of PTCDA monolayers on MgO(001)/Ag(001) samples with different Φ_sub_ presented in Figure 5b, where the shifts of both LUMO- and HOMO-related emissions to higher binding energy concomitant with the lowering of Φ_sub_ are apparent. According to Figure 5a, the first dianions can be created at Φ_sub_ = 3.3 eV, when the SUMO level crosses the Fermi level and the second Fermi level pinning regime with Φ_pin_(ICT^(2−)^) = 4.1 eV is entered. Region III is thus considered to consist of anions and dianions within the PTCDA monolayer, with the number of the latter increasing with lowering Φ_sub_, finally yielding a full PTCDA^(2−)^ monolayer.

The results of the photoemission experiments are consistent with the STM and DFT results presented in Figure 3, where PTCDA dianions were observed for an MgO(001)/Ag(001) substrate with an initial work function in the range of Φ_sub_ = 3 eV. To test if DFT can reproduce the charge transfer behavior for increasing Φ_sub_, the calculations were repeated for higher substrate work functions, which were obtained by modifying the oxygen content at the MgO/Ag interface according to procedures reported previously.^18,31,49^ The results are shown in Figure 5c, which presents the spin-unpolarized pDOS of PTCDA adsorbed on 2 ML MgO(001)/Ag(001) for substrate work functions of Φ_sub_ = 2.96, 3.67, 4.25, and 4.70 eV, respectively. The calculated final work functions (Φ_mol_) are provided in Figure 5c and are also marked in Figure 5a together with the experimental ones. We find good agreement between experimental and calculated Φ_mol_ over the entire Φ_sub_ range, suggesting that the adsorption-induced charge rearrangements, which give rise to the work function shifts, are well accounted for in the calculations. For the highest Φ_sub_ of 4.70 eV, the work function change is negligible and the point lies on the Schottky-Mott line. The LUMO is essentially above the Fermi level and hardly any charge transfer occurs. On reducing Φ_sub_ the LUMO crosses the Fermi level and at Φ_sub_ = 4.25 eV it is exactly half-filled. This situation corresponds in experiment to a monolayer of singly integer charged PTCDA molecules. The calculated Φ_mol_ (4.74 eV) agrees well with the experimentally determined Φ_pin_(ICT^(−)^) = 4.8 eV. Note that the splitting of the LUMO into a SOMO/SUMO pair is not reproduced by the calculations, which is discussed in more detail below. This is the reason why upon further reduction of Φ_sub_ the LUMO gets filled further and is still Fermi level pinned, while experimentally the vacuum level alignment regime of the singly charged molecules is observed. Only at sufficiently low work function (Φ_sub_ = 2.96 eV), when the LUMO is completely below the Fermi level and occupied by two electrons, the calculations resemble again the experimental result and the calculated and experimental Φ_mol_ agree well.

We note that the general Φ_sub_-dependent experimental trend is captured in the calculations. This is remarkable given that details such as the vacuum level alignment of the singly charged molecules and the coexistence of differently charged molecules in the Fermi level pinning regimes were not theoretically considered. This would require spin-polarized calculations in larger unit cells containing more than one PTCDA molecule and an incorporation of exchange-correlation effects beyond the semilocal treatment,^17^ which is presently computationally too demanding. Moreover, the underestimation of HOMO–LUMO gaps, which is an inherent shortcoming of semilocal DFT functionals, further prevents the appearance of vacuum level alignment in the DFT results.

**Figure 6:**
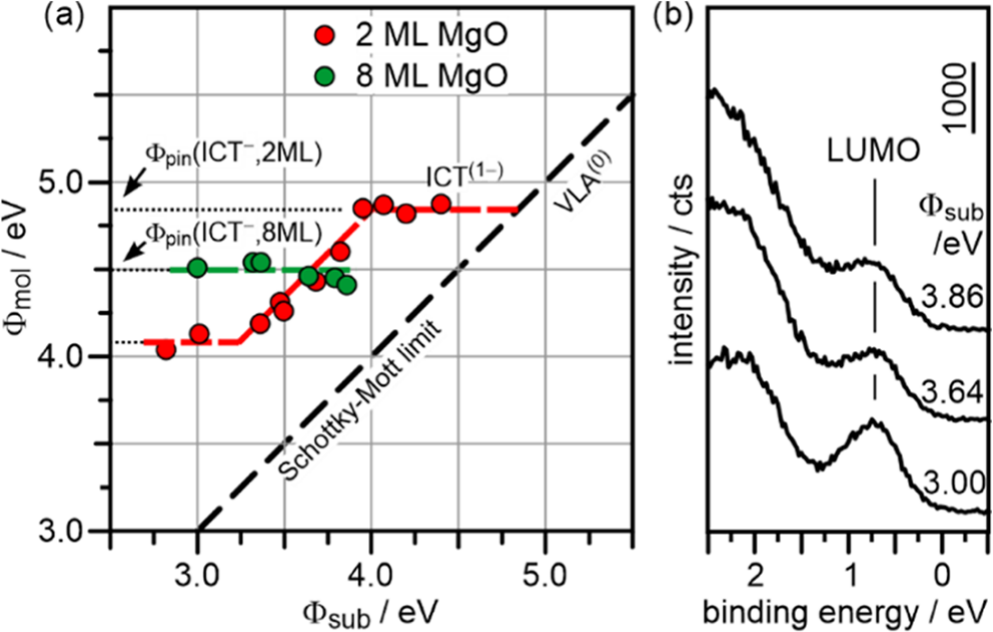
(a) Same as Figure 5a, but including the results for PTCDA on 8 ML MgO(001)/Ag(001) (green circles). The ICT^(−)^ level is lower for 8 ML MgO(001)/Ag(001) because of reduced screening on the thicker film. The extension of the ICT^(−)^ region to lower Φ_sub_ is in agreement with the capacitor model. (b) Valence-band UPS spectra of 1 ML PTCDA on 8 ML MgO(001)/Ag(001) for different Φ_sub_.

Final support for the ICT mechanism is provided by the MgO layer thickness-dependent charging of PTCDA. We have shown previously that ICT on MgO(001)/Ag(001) substrates can be described with a simple parallel plate capacitor model, where the charging-induced change of the work function ΔΦ is proportional to the areal charge density and the thickness of the dielectric thin film.^18^ When the thickness of the dielectric thin film is increased, the capacitor model predicts an increase of ΔΦ for the completely integer charged ML and an according increase of the width of the anionic Fermi level pinning regime (ICT^(1−)^ in Figure 5a). As Figure 6a shows, such an extension of the anionic Fermi level pinning regime is indeed observed for PTCDA monolayers on MgO(001) films with a nominal thickness of 8 ML. As expected in our model, a constant final work function is obtained across the whole achievable range of Φ_sub_. Note that the value of the anionic Φ_pin_ is 0.3 eV lower compared to ultrathin MgO films. The dependence of Φ_pin_ on the dielectric thickness corresponds to a change in the electron affinity. Such a dependence has been observed before for pentacene (5A) on MgO(001)/Ag(001)^18^ and indirectly for 5A and phthalocyanine on NaCl thin films.^6,50^ It can be explained by reduced screening, as the distance of the molecular layer to the metal surface increases.^51,52^ Since all data points for 8 ML thin MgO(001) films in Figure 6a correspond to the system being in the Fermi level pinning regime, we expect a constant BE of the SOMO peak, in contrast to the situation shown in Figure 5b, where the shifts of the peaks correspond to vacuum level alignment. This is indeed observed, as shown by the ARUPS spectra of PTCDA monolayers on 8 ML MgO(001)/Ag(001) with selected Φ_sub_ shown in Figure 6b.

## 4 Conclusions

Our combined experimental and computational study has shown that the integer charge transfer model, which describes the charge transfer across interfaces of weakly interacting substrate/adsorbate systems, can be extended to the double charging regime. The selection of PTCDA as molecular adsorbate with high electron affinity and MgO(001) thin films on Ag(001) as substrate, which allows the substrate work function to be tuned in a wide range, has opened the possibility to investigate the transition from single negative charging to double negative charging of PTCDA on the molecular level. Doubly negatively charged PTCDA on low work function MgO(001)/Ag(001) was identified with STM, STS and DFT. A systematic variation of the MgO(001)/Ag(001) work function and the combination of work function measurements with ARUPS data for the energy alignment of the occupied LUMO level revealed the transition from the Fermi level pinning regime of anions to the one of dianions via a vacuum level alignment of the anions. The anion vacuum level alignment regime occurs because of the SOMO-SUMO splitting of the singly occupied LUMO and the Coulomb energy that needs to be overcome to transfer a second electron into the LUMO.
